# Integrated microbiome and metabolome analysis reveals that new insight into *Radix pseudostellariae* polysaccharide enhances PRRSV inactivated vaccine

**DOI:** 10.3389/fimmu.2024.1352018

**Published:** 2024-06-26

**Authors:** Gaolin Xu, Zelong You, Yu Zheng, Qixian Feng, Shishi Luo, Lihui Xu, Songying Bao, Quanxi Wang

**Affiliations:** ^1^ Fujian Key Laboratory of Traditional Chinese Veterinary Medicine and Animal Health, Fujian Agriculture and Forestry University, Fuzhou, China; ^2^ University Key Laboratory for Integrated Chinese Traditional and Western Veterinary Medicine and Animal Healthcare in Fujian Province, Fujian Agriculture and Forestry University, Fuzhou, China; ^3^ Technical Department, Zhaofenghua Biotechnology (Fuzhou) Co. Ltd, Fuzhou, China

**Keywords:** *Enterococcus cecorum*, metabolome, inactivated PRRSV vaccine, *Radix pseudostellariae* polysaccharide, *Prevotella_copri*, taurodeoxycholic acid

## Abstract

In this study, we investigated how *Radix pseudostellariae* polysaccharide (RPP) enhances the immune response of the inactivated porcine reproductive and respiratory syndrome virus (PRRSV) vaccine through interactions with the microbiome and metabolome. We pretreated sows with 10 mg/kg body weight of RPP via drinking water for 7 days prior to intramuscular injection of the PRRSV vaccine. This significantly increased the concentrations of PRRSV GP5 protein antibody, interleukin (IL)-2, IL-4, IL-10, and interferon (IFN)-γ. Oral administration of RPP also significantly improved the abundance of beneficial bacteria in the stool, such as *Parabacteroides distasonis*, *Prevotella_copri*, *Eubacterium_sp.*, and *Clostridium_sp._CAG:226*, and decreased the levels of potentially pathogenic bacteria, such as Paraeggerthella and [Clostridium] innocuum, compared to the vaccine alone. These bacterial changes were confirmed using quantitative real-time polymerase chain reaction (Q-PCR). Moreover, RPP treatment significantly increased the blood concentrations of L-theanine, taurodeoxycholic acid (TDCA), and N-arachidonoyl proline, and decreased the levels of L-glutamine, oclacitinib, lipoxin C4, and leukotriene C5 in sows after immunization (*p<* 0.05). The concentrations of various blood metabolites were validated using sandwich enzyme-linked immunosorbent assay (ELISA), confirming the accuracy of the metabolomics data. Intriguingly, the integration of microbiome and metabolome analyses highlighted the significance of Prevotella_copri and TDCA. We consequently developed a mouse immunity model using GP5 protein and discovered that oral administration of RPP significantly enhanced the levels of GP5 protein antibodies, IL-2, IL-4, IL-10, and IFN-γ in mouse serum. It also increased the number of CD3+ and CD3+CD4+ cells in the spleen. Additionally, Prevotella_copri was administered into the large intestine via the anus for 7 days prior to the intramuscular injection of the PRRSV GP5 protein. The results demonstrated a significant increase in TDCA and GP5 antibody concentration in the mouse serum, indicating that RPP modulates *Prevotella_copri* to elevate its metabolite TDCA, thereby enhancing the GP5 antibody level. In conclusion, oral administration of 10 mg/kg RPP optimizes gut flora diversity and blood metabolites, particularly *Prevotella_copri* and TDCA, thereby improving the immune response to the inactivated PRRSV vaccine.

## 1 Introduction

Porcine reproductive and respiratory syndrome (PRRS) is one of the most economically significant diseases in the global swine industry, causing annual losses exceeding $560 million in the United States alone (1). The challenge of effectively preventing and controlling PRRS is a major bottleneck in healthy swine breeding today (2). Different regions employ various strategies for PRRS management; for instance, the European Union and the United States focus on biosecurity measures (3), whereas China and other countries rely on vaccination strategies (4). Although PRRS vaccines, including live and inactivated versions, do not offer universal protection against all strains, they are crucial in controlling the disease (5). The inactivated PRRS vaccine is safer but less effective at inducing cellular immunity compared to the live vaccine, which limits its use (6, 7). Enhancing cellular immunity in swine during the vaccination with inactivated PRRS vaccines could improve their effectiveness.

*Radix pseudostellariae* (Taizisen) (RP) is extensively cultivated and used as both a health food and a tonic herbal medicine in China and other parts of Asia (8, 9). The potential of RP to modulate human and animal immunity is garnering increasing interest (10). As a traditional Chinese medicine, RP’s polysaccharides are considered its primary active components (11, 12). Previous studies by our team have shown that *Radix pseudostellariae* polysaccharide (RPP) from fiber roots can enhance serum antibodies (IgG and IgG2b) against the OVA antigen in mice and increase transcription levels of IL-4, IL-10, and IFN-γ in the mouse spleen, suggesting its potential as a vaccine adjuvant (13). Further research is needed to explore how RPP might affect the efficacy of the inactivated PRRS vaccine.

Gut microbes and circulating metabolites have garnered significant interest. Intestinal microbes are recognized as novel virtual metabolic organs and play a crucial role in the development of numerous diseases. In this study, we investigated whether and how RPP enhances the immune effects of the inactivated PRRSV vaccine by integrating with the intestinal microbiome and serum metabolome. The findings will provide insights into the use of RPP as an effective, non-toxic, and safe immune adjuvant in traditional Chinese medicine.

## 2 Materials and methods

### 2.1 Animals and polysaccharide

Healthy, non-pregnant sows, which tested negative for PRRSV antibodies and antigens, were sourced from Datian Jinsheng Hoggery (Datian, China; **Supplementary Material 1**). Specific pathogen-free (SPF) Institute of Cancer Research (ICR) mice were acquired from the Fuzhou Wushi Experimental Animal Company. All animal experiments complied with the guidelines of the Research Ethics Committee of Zhaofenghua Biotechnology Co., Ltd, Fuzhou (approval no. LL-2022-005). The RPP, which was leached by distilled water and was extracted by chloroform:n-butyl alcohol (5:1), containing 78% polysaccharide, was purchased from Waltlet Biotechnology Co., Ltd., Lanzhou, Gansu, China (**Supplementary Material 2**).

### 2.2 Grouping and immunization

A total of 25 sows that tested negative for antigens of several viruses including swine fever, PRRSV, porcine circovirus, pseudorabies, and foot and mouth disease, and negative for PRRSV antibodies, were randomly divided into five groups, with five sows in each group. Group 1 served as the blank control group, while Group 2 received only the inactivated PRRSV vaccine. Groups 3 to 5 were treated with the inactivated PRRSV vaccine plus RPP at dosages of 10 mg/kg, 50 mg/kg, and 250 mg/kg, respectively. Groups 3–5 received RPP orally through drinking water for 7 days prior to immunization, while Groups 1 and 2 received plain drinking water as a control. On the eighth day, Groups 2–5 were vaccinated with one dose of the inactivated PRRSV vaccine (strain CH-1a, Zhaofenghua Biotechnology Co., Ltd., Fuzhou, China; 10^8.0^ TCID50/mL). A second dose was administered on the 15th day after the initial immunization, and Groups 3–5 received an additional 7 days of RPP orally before this second vaccination.

### 2.3 Sample collection

Blood and serum samples were collected from each group of sows at various times: before the first immunization, 1 week after the first and second immunizations, 2 weeks after the first and second immunizations, and 4 weeks after the second immunization.

Additionally, fresh stool samples from Groups 1–3 were collected on the seventh day post-second immunization for metagenome sequencing and Q-PCR analysis.

### 2.4 Antibody detection of PRRSV by enzyme-linked immunosorbent assay

Serum was separated from blood samples collected from five sows, and the concentration of PRRSV GP5 protein antibody was detected using a double-antibody sandwich enzyme-linked immunosorbent assay (ELISA) kit (ZCIBIO Technology Co., Ltd, Shanghai, China). Standard samples (at concentrations 6.25, 12.5, 25, 50, 100, and 200 ng/mL) were used to create the concentration standard curve (**Supplementary Material 3**). The samples were analyzed by ELISA (OD = 450 nm), and the GP5 antibody concentration was calculated based on the standard curve.

### 2.5 Cytokine transcriptional level by Q-PCR detection

Total RNAs from peripheral blood cells were extracted using the TransZol Up Plus RNA kit (ER501, TransGen, China). The complementary DNA (cDNA) was synthesized using the GoScript™ Reverse Transcription System reagent cDNA amplification kit (A5001, Promega, USA). The reverse transcription protocol included 1 µL of Oligo (dT), 0.5 µL of recombinant RNasin^®^, 2 µL of 25 mM magnesium chloride (MgCl_2_), 4 µL of GoScript™ 5 buffer, 1 µL of random primer, 1 µL of reverse transcription, 3 µL of mRNA, 1 µL of 10 mM PCR nucleotide mix, and 6.5 µL of nuclease-free water, incubated at 42°C for 15 min and 72°C for 15 min (14).

The specific primers of IL-2 (gene ID: 396868), IL-4 (gene ID: 397225), IL-10 (gene ID: 397106), and IFN-γ (gene ID: 396991) were designed by Primer Premier 15 (Premier Co., Ltd, Canada) as follows: IL-2 F: 5′-TACAGCGGAAGCACAGCAG-3′, R: 5′-CGCAGAGGTCCAAGTTCATC-3′; IL-4 F: 5′-AACGAGGTCACAGGAGAAGG-3′, R: 5′-TGGAAGCCCTACAGACAAGC-3′; IL-10 F: 5′-AATAACTGCACCCACTTCCCA-3′, R: 5′-GGTAAAACTGGATCATTTCCG-3′; IFN-γ F: 5′-CGCTACACACTGCATCTTGG-3′, R: 5′-TTCCACATCTATGCCACTTGAG-3′; and β-actin F: 5′-GAGACCTTCAACACCCCAGCC-3′, R: 5′-AATGTCACGCACGATTTCCC-3′. The mRNA levels of IL-2, IL-4, IL-10, and IFN-γ in sows were measured using Q-PCR with the GoTaq^®^ qPCR Master Mix [A6001, Promega (Beijing) Biotech Co., Ltd, USA]. The PCR conditions included 1 µL of cDNA, 6.25 µL of SYBR^®^ Premix EX Taq™, 0.25 µL of primer F, 0.25 µL of primer R, and 12.25 µL of ddH_2_O. The thermal cycling conditions were 95°C for 5 min, followed by 45 cycles of denaturation at 95°C for 10 s, annealing at 60°C for 10 s, and extension at 72°C for 10 s. The reaction was finalized by an extension at 72°C for 10 min and storage at 4°C. β-Actin served as the reference gene for calculating the relative expression of target genes using the 2^−ΔΔCt^ method (15).

### 2.6 Metagenomics revealed the candidate microorganisms

To investigate whether RPP influenced gut microbiota and enhanced immunity in sows, fecal samples were collected using a cotton swab for metagenomic sequencing. DNA was extracted using a MagPure soil DNA KF kit (D6356-F-96-SH, Magen, Guangzhou, China). The DNA was then fragmented using S220 Focused-ultrasonicators (Covaris, USA) and purified with Agencourt AMPure XP beads (Beckman Coulter Co., USA). Libraries were prepared using the TruSeq nano DNA LT sample preparation kit (Illumina, San Diego, CA, USA). Metagenomic sequencing and analysis were carried out by OE Biotech Co., Ltd. (Shanghai, China) (16).

Metagenome assembly was performed using MEGAHIT (v1.1.2) (17, 18) (https://github.com/voutcn/megahit) after obtaining valid reads. The open reading frames (ORFs) of assembled scaffolds were predicted with the Prokaryotic dynamic programming gene-finding algorithm (Prodigal) (v2.6.3) (https://codeload.github.com/hyattpd/Prodigal/tar.gz/v2.60) (19) and translated into amino acid sequences. Clean reads from each sample were aligned against a non-redundant gene set (95% identity) using Bowtie 2 (v2.2.9) (http://bowtie-bio.sourceforge.net/bowtie2/index.shtml). Gene abundance in each sample was quantified accordingly.

Taxonomic classifications were derived from the NR library’s taxonomy database, and species abundance was calculated based on gene abundance. Abundance profiles were constructed at each taxonomic level—domain, kingdom, phylum, class, order, family, genus, and species—by performing abundance statistics. The gene set’s representative amino acid sequences were annotated using databases such as NR, Kyoto Encyclopedia of Genes and Genomes (KEGG) (20, 21), EggNOG (http://eggnog6.embl.de/) (22), Universal Protein (UniProt) (https://www.uniprot.org/), and Gene Ontology (GO) (http://geneontology.org/) with an e-value threshold of 1e-5 (23). Gene sets were compared with the CAZy database (http://www.cazy.org/) to identify and quantify carbohydrate-active enzymes based on gene abundances.

The original sequencing data for this metagenome study are available in the National Center for Biotechnology Information (NCBI) Sequence Read Archive, under accession numbers AMN38353060, SAMN38353061, and SAMN38353062.

The abundance of bacteria was reconfirmed using qPCR again. The special primers of *Prevotella_sp._P4-119* (F: 5′-ATTTTGTATATTTGGGGCCTA-3′, R: 5′-TATGCTATCTTTGGCACGTCA-3′), *Prevotella_sp._P5-50* (F: 5′-TTCTCATTTGAACCGAATCCG-3′, R: 5′-TGTGATTTGCATTTTACCGAT-3′), and *Bacterium_P201* (F: 5′-CCACATTGTTGGTCACCGAGA-3′, R: 5′-CCGAATTTTGCCGCTAACGAA-3′) were designed. Bacterial DNA in feces was extracted by the TransZol Up Plus RNA kit (ER501, TransGen, China). The Q-PCR reaction conditions were referred to the cytokine detection section. Eub was used as the reference gene (F: 5′-AGAGTTTGATCCTGGCTC-3′, R: 5′-TGCTGCCTCCCGTAGGAGT-3′).

### 2.7 Metabolomics analysis on the key metabolins

To investigate the effect of RPP on sow metabolism, we utilized a liquid chromatography–mass spectrometry (LC-MS) system, comprising a Dionex U3000 UHPLC from Thermo Fisher Scientific, USA, and a QE plus high-resolution mass spectrometer, also from Thermo Fisher Scientific, USA. We randomly selected three peripheral blood samples each from the control (blank control group and PRRSV inactivated vaccine control group) and low-dose groups for non-targeted metabolomic analysis. The samples were analyzed using both positive and negative ion scanning modes, and the data were processed with Progenesis QI metabolomics software (version 2.3, Munich, Bavaria, Germany) (24). The study data have been stored in the OMIX database at the China National Center for Bioinformation/Beijing Institute of Genomics, Chinese Academy of Sciences (https://ngdc.cncb.ac.cn/omix: accession No. OMIX005274).

Furthermore, the concentrations of L-glutamine, leukotriene C5, L-theanine, and TDCA in the peripheral blood of Group 1, 2, and 3 sows were measured again using ELISA, conducted by Yuanxin Biotechnology Co., Ltd, Shanghai, China.

### 2.8 Integrated microbiome and metabolome analysis

This study also explores whether there is a link between changes in gut microbiota and peripheral blood metabolites following oral RPP administration, by employing Spearman correlation analysis. This analysis assesses the relative abundance of gut microbiota and the corresponding metabolite response intensities, establishing a relationship based on a one-to-one correspondence between samples. This approach helps to define the interaction between gut microbiota and blood metabolites in sows treated with RPP as an immune adjuvant (25). The correlation coefficient is calculated by the computational formula as $r_{s}=\frac{\sum \left(R_{X}-\bar{R_{X}}\right)\left(R_{Y}-R_{Y}\right)}{\sqrt{{\sum \left(R_{X}-\bar{R_{X}}\right)}^{2}{\sum \left(R_{Y}-\bar{R_{Y}}\right)}^{2}}}=\frac{\sum R_{X}R_{Y}-\frac{\left(\sum R_{X}\right)\left(\sum R_{Y}\right)}{n}}{\sqrt{\left(\sum R_{X}^{2}-\frac{{\left(\sum R_{X}\right)}^{2}}{n}\right)\left(\sum R_{Y}^{2}-\frac{{\left(\sum R_{Y}\right)}^{2}}{n}\right)}}$ . The correlation is shown as the cluster heatmap. The correlation coefficient ranges from [−1,1], the positive number means positive correlation, and the negative number means negative correlation. If the absolute value is the greater, the correlation is higher (25).

### 2.9 Immunity effect of RPP assists PRRSV GP5 protein in mice

Seventy-five 3-week-old ICR mice (20 ± 2 g), sourced from Wushi Experimental Animal Center, Fujian, China, were acclimated for 7 days and then randomly divided into five groups, each with 15 animals. The first group served as the blank control, while the second group was the GP5 immune control. Groups 3, 4, and 5 were treated with GP5 (**Supplementary Material 4**) and varying doses of RPP: 10 mg/kg, 50 mg/kg, and 250 mg/kg, respectively. Before immunization, Groups 3–5 received gastric gavage with distilled water and their respective RPP doses. At the time of immunization, all groups except the first one received 0.2 mL of the GP5 protein solution subcutaneously, each dose containing 100 µg of GP5 protein (≥99.9% antigen content). Mice had free access to food and water throughout the study.

Blood was collected from the orbital venous plexus of mice in all groups both pre-immunization and 2 weeks post-immunization. Serum was separated for the measurement of blue ear GP5-specific antibodies and cytokines IL-2, IL-4, IL-10, and IFN-γ, using a double antibody sandwich ELISA technique (ZCIBIO Technology Co., Ltd, Shanghai, China).

The spleens from mice in Groups 1, 2, and 3 were collected 2 weeks post-immunization. The spleen cell suspension was prepared and centrifuged at 1,500 rpm for 5 min. After treating with red blood cell lysates for 8 min, the suspension was centrifuged again under the same conditions. Cells were resuspended in phosphate-buffered saline (PBS), forming a suspension of single spleen cells. These cells were then incubated with fluorescently labeled monoclonal antibodies against CD3+ (PE), CD4+ (PerCP), and CD8+ (APC) at 4°C for 30 min (BioLegend, San Diego, CA, USA). The cell suspension was subsequently analyzed by flow cytometry to determine the proportions of T lymphocyte subsets (CD3+, CD3+CD4+, CD3+CD8+, and CD3+CD4+CD8^+^) (26).

### 2.10 Oral administration of *Prevotella_copri* on mice

Twenty 3-week-old ICR mice were randomly divided into two groups: a blank control group and a Prevotella_copri (DSM18205, MingzhouBio, Ningbo, China) treatment group, with each group comprising 10 mice. The Prevotella_copri treatment group received oral administration of Prevotella_copri (OD_600nm_ = 1, 0.2 mL per mouse) for 10 days. Serum and feces were collected from both groups for analysis. The concentration of TDCA in serum was determined by ELISA, and the abundance of Prevotella_copri in feces was measured using Q-PCR.

Fifty 3-week old ICR mice were randomly divided into a blank control group, a GP5 (0.2 mL, 100 μg/mouse) subunit vaccine control group, a GP5 + RPP (10 mg/kg) coprocessing group, a GP5 + *Prevotella_copri* coprocessing group, and a GP5 + RPP (10 mg/kg) + *Prevotella_copri* coprocessing group, with 10 mice in each group. Seven days before immunization, while mice were subjected to oral administration with sterile PBS treatment in blank control and vaccine control groups, others were orally administered with RPP and/or *Prevotella_copri* (OD_600nm_ = 1, 0.2 ml/mouse). Serum and feces were collected the day before immunization and 14 days after immunization. The concentrations of GP5-specific antibody, IL-2, IL-4, and TDCA in the serum were determined by ELISA. The relative expression of *Prevotella copri* was determined by QPCR.

### 2.11 Statistical analysis

Data were analyzed using GraphPad Prism 9 (GraphPad Software, San Diego, CA, USA) through one-way ANOVA for data processing and statistical analysis. A *p*-value of less than 0.05 was considered statistically significant.

## 3 Results

### 3.1 Oral administration of RPP promotes anti-Gp5 antibody concentration in sows vaccinated with inactivated PRRSV vaccine

**Figure 1:**
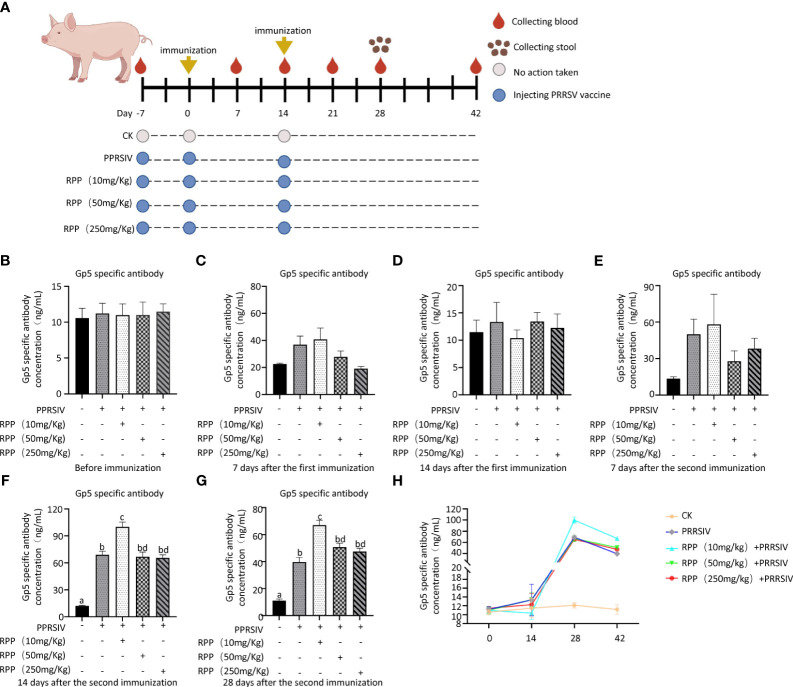
RPP promoted the antibody concentration of inactivated PRRSV vaccine. A total of 25 sows, all with the same pregnancy parity and free from specific antigens (ASF virus, swine fever virus, porcine reproductive and respiratory syndrome virus, pseudorabies virus, and circovirus), were randomly divided into five groups. Group 1 served as the blank control. Group 2 received only the inactivated PRRSV vaccine. Groups 3, 4, and 5 were treated with the inactivated PRRSV vaccine combined with RPP at dosages of 10 mg/kg, 50 mg/kg, and 250 mg/kg, respectively. Groups 3–5 received RPP orally via drinking water for 7 days before the first and second immunizations, whereas Groups 1 and 2 were given plain water as a control. The immunization plan was present in **(A)** Serum samples were collected the day before immunization **(B)**, 1 week after the first immunization **(C)**, 2 weeks after the first immunization **(D)**, 1 week after the second immunization **(E)**, 2 weeks after the second immunization **(F)**, and 4 weeks after the second immunization **(G)**. The antibody growth and decline pattern was shown in **(H)** The antibody concentration of GP5 was measured using a double antibody sandwich ELISA. All data were presented as the mean ± standard deviation (SD) of three independent experiments. Significance between the treatments was determined by independent-samples ANOVA. Statistical significance was determined using single-factor ANOVA with SPSS software (Version 20.0), where values with different superscript letters indicate significant differences (p< 0.05).

The study results indicated that the antibody concentrations of GP5 protein were uniformly negative across all groups prior to vaccination with the inactivated PRRSV vaccine (**Figure 1A**), showing no significant differences (**Figure 1B**). These concentrations remained negative up to the 14th day after the first immunization (**Figures 1C, D**) and on the 7th day following the second immunization (**Figure 1E**). However, on the 14th day after the second immunization, Group 3, treated with 10 mg/kg RPP, exhibited a significant increase in antibody concentrations compared to other groups (*p<* 0.05) (**Figure 1F**). Twenty-eight days after the second immunization, antibody concentrations of GP5 protein in Groups 2–5 decreased compared to those on the 14th day after the second immunization. However, antibody concentrations in Group 3, treated with 10 mg/kg RPP, were significantly higher than in Group 2, which received only the inactivated PRRSV vaccine (**Figures 1G, H**). These results suggest that oral administration of 10 mg/kg RPP can significantly enhance the immunogenic effect of the inactivated PRRSV vaccine in sows.

### 3.2 Oral administration of RPP promotes the cellular immune function in sows vaccinated with inactivated PRRSV vaccine

**Figure 2:**
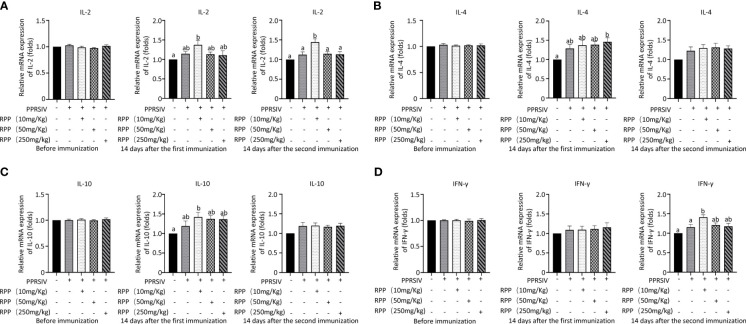
Cytokine transcriptional level by Q-PCR detection. At 0 days (left), 1 week (middle), and 2 weeks (right), the mRNA levels of IL-2 **(A)**, IL-4 **(B)**, IL-10 **(C)**, and IFN-γ **(D)** in serums of sows were detected by Q-PCR. All data were presented as the mean ± SD of three independent experiments. Significance between the treatments were determined by independent-samples ANOVA. Statistical significance was determined using single-factor ANOVA with SPSS software (Version 20.0), where values with different superscript letters indicate significant differences (p< 0.05).

The mRNA levels of IL-2, IL-4, IL-10, and IFN-γ were then evaluated across all groups. Initially, there were no significant differences in IL-2 mRNA levels among the groups before vaccination with the inactivated PRRSV vaccine (**Figure 2A**, left). Fourteen days after the first immunization, IL-2 mRNA levels had significantly increased in Group 3 compared to Group 1 (**Figure 2A**, middle). Similarly, on the 14th day following the second immunization, IL-2 mRNA levels were significantly higher in Group 3 than in Group 1 (**Figure 2A**, right). This indicates that oral administration of 10 mg/kg RPP can significantly boost IL-2 mRNA levels.

The mRNA levels of IL-4 were measured, and results showed a significant increase on the 14th day after vaccination with the inactivated PRRSV vaccine in Group 5 compared to Group 1. This suggests that RPP has a minimal effect on IL-4 in sows (**Figure 2B**). Additionally, we assessed the impact of RPP on IL-10 mRNA levels and observed a significant increase in Group 3 on the 14th day post-vaccination. Oral administration of 10 mg/kg RPP significantly enhanced IL-10 mRNA levels (**Figure 2C**). Lastly, IFN-γ mRNA levels were notably higher in Group 3 on the 14th day after vaccination compared to Groups 1 and 2 (**Figure 2D**). These findings indicate that 10 mg/kg of oral RPP enhances cellular immune function in sows vaccinated with the inactivated PRRSV vaccine.

### 3.3 Oral administration of RPP-optimized intestinal microbiota of sows vaccinated with inactivated PRRSV vaccine

**Figure 3:**
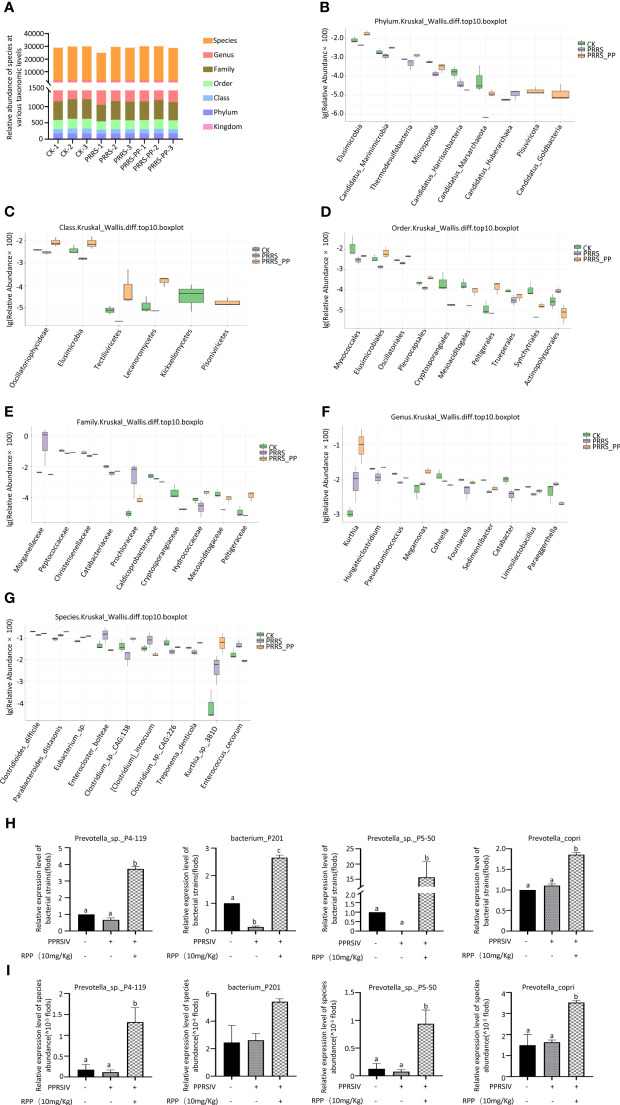
Distribution of the intestinal microflora by metagenomics. Fresh fecal samples were collected aseptically on the 7th day following the second immunization with the inactivated PRRSV vaccine and subjected to metagenomic sequencing. The abundance of species at taxonomic levels including phylum, class, order, family, genus, and species were reported in section **(A)**. The top 10 species with significant differences at various taxonomic levels were detailed in sections **(B)** (phylum), **(C)** (class), **(D)** (order), **(E)** (family), **(F)** (genus), and **(G)** (species), based on p-values accompanied by box plots. The abundance of four species were redetermined by Q-PCR, their results were present on **(H)**, while results present on **(I)** were determined by Metagenomics The data from Q-PCR were presented as the mean ± SD of three independent experiments. Significance between the treatments were determined by independent-samples ANOVA. Statistical significance was determined using single-factor ANOVA with SPSS software (Version 20.0), where values with different superscript letters indicate significant differences (p< 0.05).

To determine the role of intestinal microbiota in immune function regulation, our team analyzed fecal samples from three groups (1, 2, and 3) using metagenomics. **Figure 3A** (**Supplementary Material 5**) presents significant data variations. We identified the top 10 species at various taxonomic levels including phylum (**Figure 3B**), class (**Figure 3C**), order (**Figure 3D**), family (**Figure 3E**), genus (**Figure 3F**), and species (**Figure 3G**) (**Figure 3**).

Interestingly, the abundance of *Peptococcaceae*, *Christensenellaceae*, and *Catabacteriaceae* at the family level was significantly improved in Group 3 (*p<* 0.05; **Figure 3E**), the abundance of *Limosilactobacillus*, *Megamonas*, and *Fournierella* was significantly increased at the genus level (*p<* 0.05) (**Figure 3E**), and the abundance of *Eubacterium* sp., *Parabacteroides_distasonis*, and *Clostridium_sp._CAG:226* was significantly promoted at the species level (**Figure 3G**).

We assessed the accuracy of metagenome data using Q-PCR with specific primers. The results showed that the trends of species such as Prevotella_sp._P4-119, Bacterium_P201, and Prevotella_sp._P5-50 were in line with the metagenomic sequencing method (**Figure 3H, I**). This suggests that oral administration of RPP at 10 mg/kg enhances the growth of beneficial gut bacteria in sows.

### 3.4 Oral administration of RPP-regulated peripheral blood metabolins of sows vaccinated with the inactivated PRRSV vaccine

**Figure 4:**
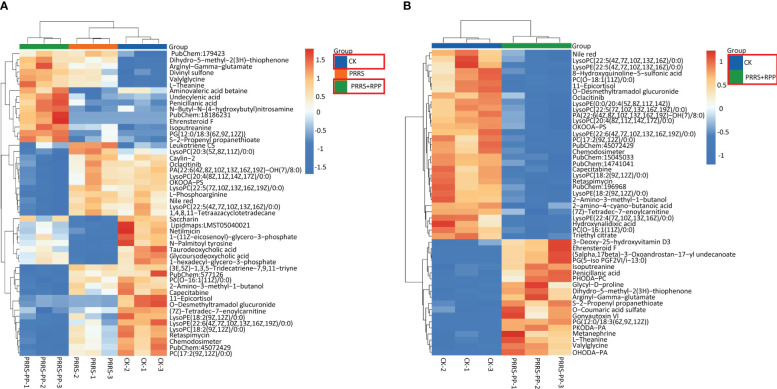
Different metabolins were screened by metabolomics. Serum samples were collected from sows in Groups 1, 2, and 3 for blood metabolomic sequencing on the 7th day after the second immunization with the inactivated PRRSV vaccine. Hierarchical clustering was performed on the expression levels of all significantly different metabolites, and the top 50 were ranked according to their variable importance in projection (VIP) scores. The metabolites were presented for comparisons among Groups 1, 2, and 3 (section **A)** and between Groups 1 and 2 (section **B)**, respectively. The horizontal axis denotes the sample names, while the vertical axis represents the differential metabolites. The color gradient from blue to red indicates the expression abundance of metabolins; the more the red color the figure shows, the higher the abundance of differential metabolins.

**Figure 5:**
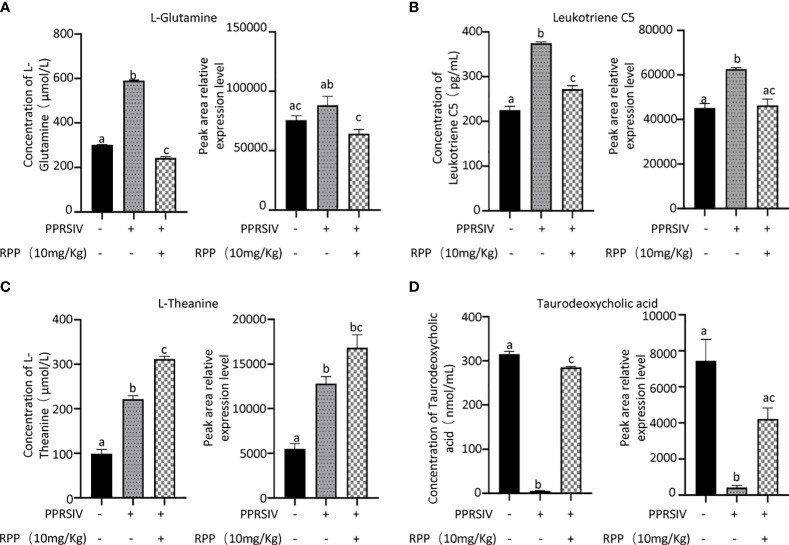
Abundance of bacteria in the feces of sows was reconfirmed by Q-PCR. Serum samples from sows in Groups 1, 2, and 3 were analyzed using a double antibody sandwich ELISA to determine the concentrations of various compounds. The results included L-glutamine (section **A**, the left side showing ELISA validation and the right side showing high-throughput sequencing results), Leukotriene C5 (section **B**), L-theanine (section **C**), and taurodoxycholic acid (section **D**). Each section presents ELISA validation results on the left and high-throughput sequencing results on the right, highlighting the comparison of methodologies. All data were presented as the mean ± SD of three independent experiments. Significance between the treatments was determined by independent-samples ANOVA. Statistical significance was determined using single-factor ANOVA with SPSS software (Version 20.0), where values with different superscript letters indicate significant differences (p< 0.05).

The beneficial gut flora may release or regulate metabolites that enhance the host’s immune function. In this study, we aimed to explore the spectrum of different metabolites in the peripheral blood of sows. Blood samples from Groups 1–3 were collected for sequencing and functional analysis through metabolomics. The top 50 differing metabolites among these groups are displayed in **Figure 4** (**Supplementary Material 6**). The total number of metabolites was 133 between Group 2 and Group 1, 164 between Group 3 and Group 2, 146 between Group 3 and Group 1, and 314 across all three groups (**Table 1**). We observed that oral administration of RPP at 10 mg/kg significantly increased the levels of L-theanine, taurodeoxycholic acid, and N-arachidonoyl proline, and markedly downregulated the abundance of L-glutamine, oclacitinib, lipoxin C4, and leukotriene C5 (*p<* 0.05). We reconfirmed the abundance of four different metabolites—L-glutamine, leukotriene C5, L-theanine, and taurodeoxycholic acid—using sandwich ELISA. The results demonstrated that the trends in these metabolites’ variations were consistent between the metabolomics sequencing method and ELISA (**Figures 5A–D**). These findings suggest that oral administration of RPP at 10 mg/kg optimizes the secretion of sow metabolites.

**Table 1:** Comparison of the number of differential metabolins in different groups.

Groups	Total different metabolins	Upregulated different metabolins	Downregulated different metabolins
Group 2 vs. Group 1	133	61	72
Group 3 vs. Group 2	164	79	85
Group 3 vs. Group 1	146	75	71
Group 3 vs. Group 2 vs. Group 1	314	314	0

### 3.5 Integrated microbiome and metabolome analysis reveals new insight into immunity improvement of inactivated PRRSV vaccine by RPP

**Figure 6:**
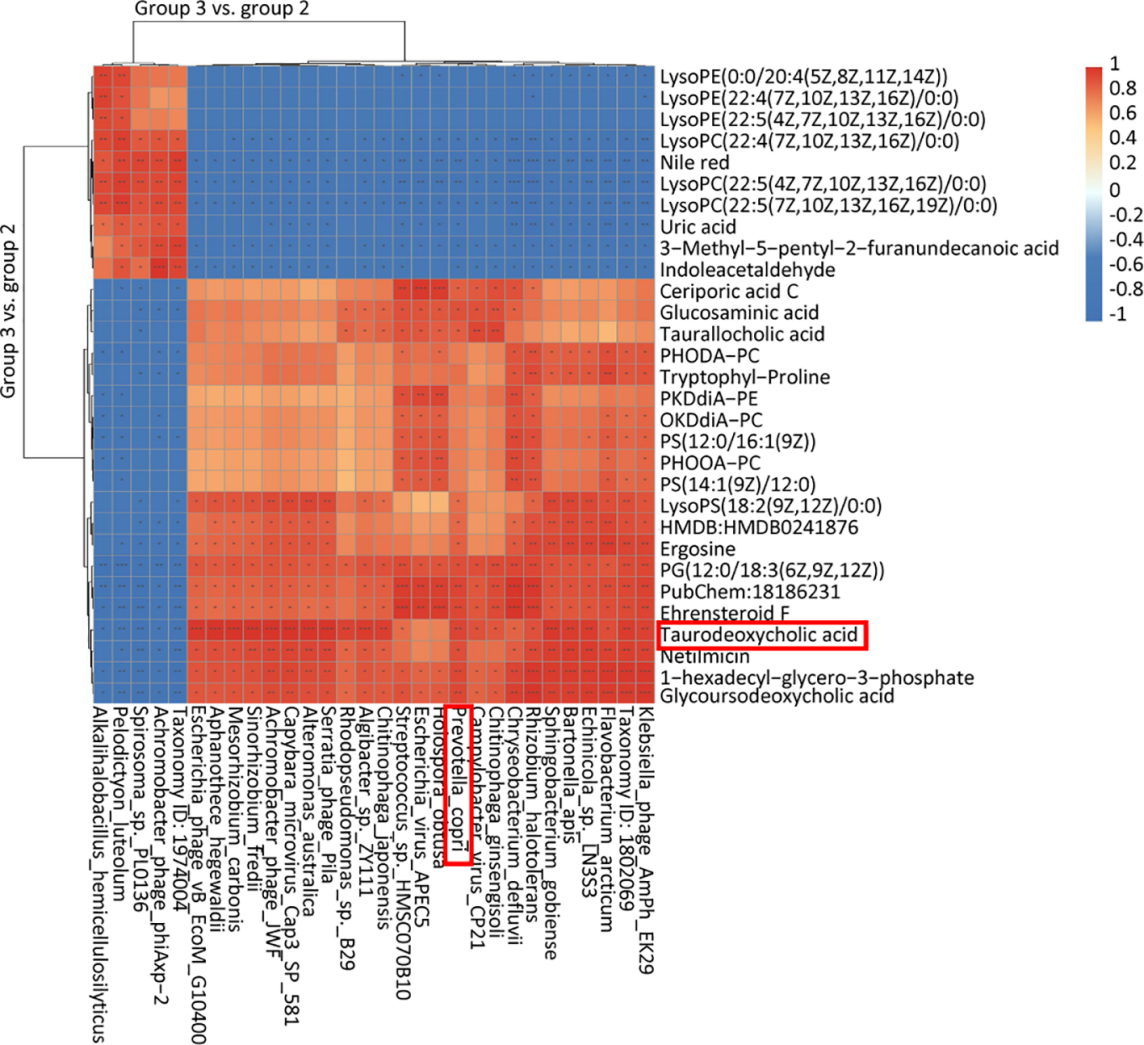
Heatmap of the top 30 association analysis between differential flora and differential metabolins in Group 3 vs. Group 2.

We explored the connection between differential flora and metabolites following oral administration of RPP. The relationships between the relative abundance of gut microbial populations and the corresponding metabolite response intensities were analyzed using the Spearman method. The results from the top 30 associations between differential flora and metabolites in Group 3 vs. Group 2 are shown in **Figure 6**. Specifically, we focused on the abundance of TDCA, which exhibited a significant positive correlation with Prevotella_copri and Rhodopseudomonas_sp._B29 (*p<* 0.05). Ceriporic acid C was positively correlated with Prevotella_copri, while uric acid showed a negative correlation with both Prevotella_copri and Sinorhizobium_fredii. This suggests that beneficial gut bacteria and their metabolites, induced by the oral administration of RPP, play a crucial role in enhancing the immune function of sows.

### 3.6 *Prevotella_copri* improved the concentration of TDCA in the peripheral blood of mice

Our studies demonstrated that the oral administration of RPP enhances the effectiveness of the inactivated PRRSV vaccine in sows. This enhancement is closely associated with an optimized gut microbiota, specifically *Prevotella_copri*, and its metabolites such as TDCA, which are induced by RPP.

**Figure 7:**
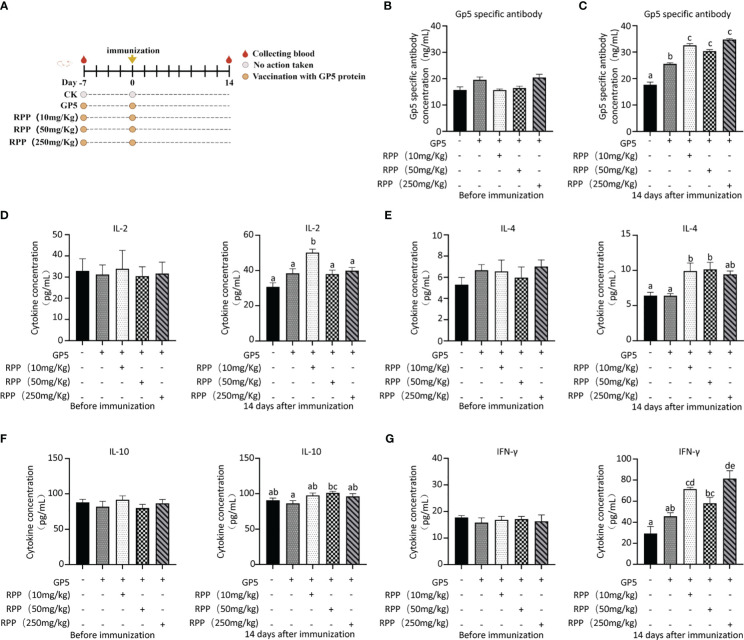
RPP improved the immune effect in mice. Seventy-five 3-week-old ICR mice (20 ± 2 g) were randomly divided into five groups after a 7-day adaptation period. These groups included the GP5 immune control group, and three treatment groups receiving GP5+RPP at doses of 10 mg/kg, 50 mg/kg, and 250 mg/kg, respectively. Starting 7 days before the first immunization, all groups except the CK and GP5 immune control groups received gastric lavage with double-distilled water and appropriate doses of RPP. For the first immunization, Group 1 received 0.2 mL of sterile PBS subcutaneously in the back, while the remaining groups received 0.2 mL of GP5 (100 µg) protein solution. The immunization plan was present **(A)**. Blood samples were collected from each group before immunization **(B)** and 2 weeks after immunization **(C)**, and serum was separated. The GP5-specific antibody was examined using ELISA. The concentration of IL-2 (**D**, left 1 pre-immunization, right 1 post-immunization 2 weeks), IL-4 (**E**, left 1 pre-immunization, right 1 post-immunization 2 weeks), IL-10 (**F**, left 1 pre-immunization, right 1 post-immunization 2 weeks), and IFN-γ (**G**, left 1 pre-immunization, right 1 post-immunization 2 weeks) in the serum was determined by ELISA. All data were presented as the mean ± SD of three independent experiments. Significance between the treatments was determined by independent-samples ANOVA. Statistical significance was determined using single-factor ANOVA with SPSS software (Version 20.0), where values with different superscript letters indicate significant differences (p< 0.05).

**Figure 8:**
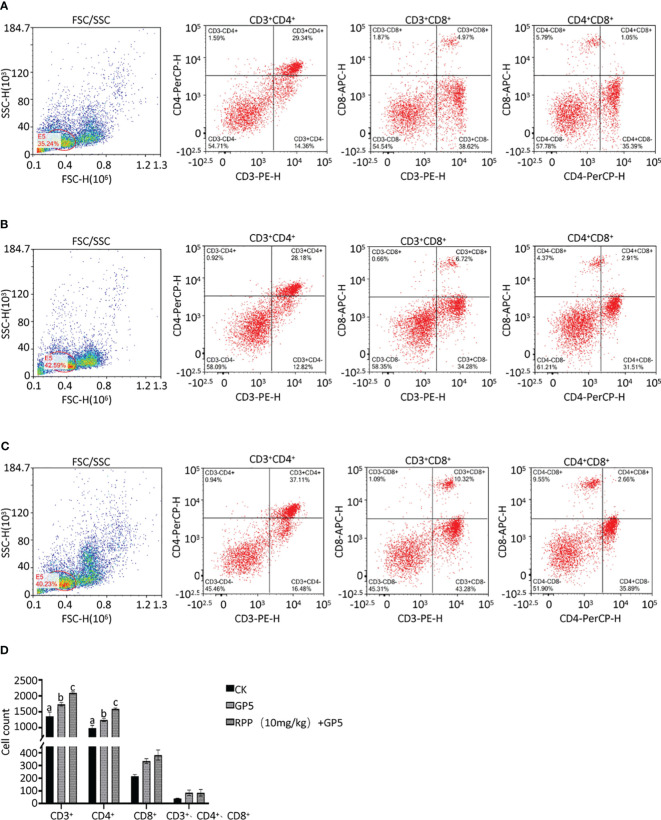
T lymphocyte subset sorting by a flow cytometer. Spleens from mice in Groups 1, 2, and 3 were harvested 2 weeks post-immunization, processed into single cells, and stained with fluorescently-labeled monoclonal antibodies against CD3+ (PE), CD4+ (PerCP), and CD8+ (APC). These cell suspensions were analyzed using flow cytometry to determine the numbers of T lymphocyte subsets in Group 1 (**A**, left CD3^+^CD4^+^, middle CD3^+^CD8^+^, and right CD4^+^CD8^+^), Group 2 (**B**, left CD3^+^CD4^+^, middle CD3^+^CD8^+^, and right CD4^+^CD8^+^), and Group 3 (**C**, left CD3^+^CD4^+^, middle CD3^+^CD8^+^, and right CD4^+^CD8^+^). The statistical analysis results are presented in **(D)**. All data were presented as the mean ± SD of three independent experiments. Significance between the treatments was determined by independent-samples ANOVA. Statistical significance was determined using single-factor ANOVA with SPSS software (Version 20.0), where values with different superscript letters indicate significant differences (p< 0.05).

Owing to increased biosafety measures in pig farms responding to African swine fever (ASF), we chose mice as a model to explore the interaction between *Prevotella_copri* and TDCA. After administering RPP at a dose of 10 mg/kg orally before the intramuscular inoculation of the GP5 subunit vaccine (**Figure 7A**), significant improvements were observed in antibody levels against GP5, as well as in the concentrations of IL-2, IL-4, and IFN-γ, compared to control groups (**Figures 7B–G**). Flow cytometry analysis of T lymphocyte subsets revealed that pretreatment with RPP increased the numbers of CD3+ T lymphocytes and CD3+CD4+ T lymphocytes, and regulated CD3+CD8+ T lymphocytes and CD3+CD4+CD8+ T lymphocytes (**Figures 8A–D**). These findings confirm that pretreatment with RPP enhances immune responses in mice, making them a suitable model for this research.

**Figure 9:**
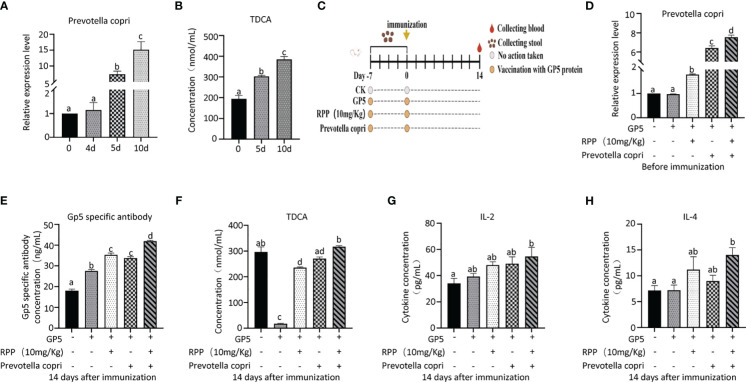
Oral administration of Prevotella_copri improved TDCA in mice. Twenty 3-week-old ICR mice (20 ± 2 g) were randomly divided into a blank control group and an experimental group, with 10 mice in each group. The control group received a standard diet, while the experimental group was administered Prevotella_copri (0.2 mL/mouse, centrifuged and resuspended in PBS with an OD_600nm_ reading) daily. After 10 days of treatment, the relative expression levels of Prevotella_copri in feces were assessed using Q-PCR **(A)**, and serum levels of TDCA were measured by ELISA **(B)**. Then, 50 3-week old ICR mice were randomly divided into a blank control group, GP5 (0.2 mL, 100 μg/mouse) a subunit vaccine control group, a GP5 + RPP (10 mg/kg) coprocessing group, GP5 + Prevotella_copri coprocessing group, and a GP5 + RPP (10 mg/kg) + Prevotella_copri coprocessing group, with 10 mice in each group. The GP5 immunizaiton plan was present in **(C)** After 14 days of immunization, the relative expression levels of Prevotella_copri in feces were assessed using Q-PCR **(D)**, and serum levels of GP5 **(E)**, TDCA **(F)**, IL-2 **(G)**, and IL-4 **(H)** were measured by ELISA. All data were presented as the mean ± SD of three independent experiments. Significance between the treatments was determined by independent-samples ANOVA. Statistical significance was determined using single-factor ANOVA with SPSS software (Version 20.0), where values with different superscript letters indicate significant differences (p< 0.05).

After administering *Prevotella_copri* orally to mice for 7 days, we observed a significant increase in TDCA concentration in the peripheral blood compared to the control group, suggesting that *Prevotella_copri* treatment can elevate TDCA levels in the blood (**Figures 9A, B**).

The results showed that the concentrations of *Prevotella_copri* in feces of RPP and/or *Prevotella copri* treatment groups on the day before immunization with GP 5 subunit vaccine group were increased significantly, compared with the blank group or the vaccine control group (*p*< 0.05) (**Figures 9C, D**). The concentrations of *Prevotella_copri* in feces of mice treated with RPP were significantly increased, compared with only vaccine treatment group (*p<* 0.05), and they were increased significantly in mice treated with RPP and *Prevotella_copri* before immunization, compared with mice only treated with RPP before immunization (*p<* 0.05). Fourteen days after immunization with GP5 subunit vaccine, the specific antibody concentration of the GP5 was significantly improved in mice treated with RPP and/or *Prevotella_copri*, compared with the blank control and only vaccine treatment group (*p*< 0.05) (**Figure 9D**). The specific antibody concentration of GP5 was significantly increased in mice that were treated with RPP and *Prevotella copri* before immunization with GP5 subunit vaccine, compared with the other four groups (*p*< 0.05) (**Figure 9E**). The concentration of TDCA in the peripheral blood was significantly decreased in only GP5 subunit vaccine treatment mice, but it was significantly increased in mice that were treated with RPP before immunization (*p<* 0.05) (**Figure 9E**). The result also showed that the concentration of TDCA was significantly improved in mice that were treated with RPP and *Prevotella copri*, compared with the other four groups (*p<* 0.05) (**Figure 9F**). Furthermore, the concentration of IL-2 and IL-4 in serum was significantly enhanced in mice treated with RPP and *Prevotella_copri* before immunization with GP5 subunit vaccine, compared with the other four groups (*p*< 0.05) (**Figures 9G, H**).

The above results revealed that *Prevotella_copri* could be increased by oral administration of RPP before GP5 subunit vaccine, which further promoted the concentration of TDCA, IL-2, and IL-4 to improve the specific antibody concentration of the GP5.

## 4 Discussion

PRRS has caused significant economic losses to the global swine industry. Developing effective prevention and control measures for PRRS remains a substantial challenge. Vaccination remains a cost-effective method for preventing PRRSV infection, though controlling the disease is still challenging. In recent years, inactivated PRRSV vaccines have garnered attention for their safety compared to live attenuated vaccines (27, 28). However, these vaccines often provide incomplete cellular immunity, resulting in suboptimal immune responses (29). Research has shown that traditional Chinese medicine and its extracts can enhance immunity and modulate immune responses (30). Peng et al. reported that moderate doses of Taishan Pinus massoniana pollen polysaccharide, used as an adjuvant with PRRSV GP5, effectively prevent and control highly pathogenic PRRSV (31). Moreover, combining tylvalosin with Poria cocos polysaccharides offers a promising approach to PRRS treatment (32). Astragalus polysaccharide has been shown to modulate immune responses in cells exposed to PRRSV or classical swine fever virus (33). In our study, we demonstrated that oral administration of 10 mg/kg RPP for 7 days before administering the inactivated PRRSV vaccine significantly enhanced immune responses in sows including cellular responses and humoral immune responses. The current results of RPP treatment before administering the inactivated PRRSV vaccine revealed that traditional Chinese medicine polysaccharide could promote the cellular immune response of animals and RPP could improve the incomplete cellular immunity of the inactivated PRRSV vaccine.

Polysaccharides from Chinese medicinal herbs, such as the Taishan Pinus massoniana pollen polysaccharide, have shown to significantly enhance immunity and serve as effective immune adjuvants (34). Specifically, they improve the production of anti-HP-PRRSV neutralizing antibodies in pigs when co-administered with a recombinant HP-PRRSV glycoprotein 5 subunit (31). Astragalus polysaccharides have been found to boost immune responses and enhance the immunological function of vaccines against Newcastle disease and avian influenza in chickens (35). These polysaccharides exert a strong immunomodulatory effect, enhancing cytokine expression and the activation of NK cells, CD4+, and CD8+ T cells (36). Previous studies have demonstrated that red pine polysaccharide (RPP) can increase cytokine levels such as IL-2, IL-4, IL-10, and IFN-γ, as well as enhance the number and activity of CD4+ and CD8+ T cells and NK cells in mice (13). Our current research confirms that pretreatment with RPP before administering an inactivated PRRSV vaccine through intramuscular injection enhances antibody production and cytokine levels in sows, supporting the vaccine’s immunogenicity, which indicated that RPP would be a potential immune adjuvant.

To further explore the mechanism by which RPP enhances immunity to inactivated PRRSV vaccines, our team investigated the fecal flora and blood metabolites. The findings indicated that pretreatment with oral RPP before administering the inactivated PRRSV vaccine intramuscularly optimized the microflora profile. This optimization included significant improvements in the abundance of *Eubacterium_sp.*, *Parabacteroides_distasonis*, and *Clostridium_sp._CAG:226* in sow feces and increased concentrations of L-theanine, taurodeoxycholic acid, and N-arachidonoyl proline in the serum. Spearman’s correlation analysis further revealed a positive correlation between the abundance of Prevotella_copri and the concentration of TDCA. This correlation piqued our interest. We hypothesized that the pretreatment with orally administered RPP boosts the immune response to the inactivated PRRSV vaccine, an effect closely linked to Prevotella_copri and its metabolite TDCA. The gut microbiota, both in humans and in animals, is recognized as a crucial factor in regulating both adaptive and innate immunity. This regulation occurs through the release of metabolites that enter the bloodstream (37).

In the gastrointestinal tract, epithelial cells are pivotal as they act as guardians by conveying crucial information to immune cells located in the lamina propria through the innate immune system’s pattern recognition receptors (PRRs), such as toll-like receptors (TLRs) and NOD-like receptors (NLRs). Additionally, the integrity of the gut barrier is maintained through intricate communication between gut microbes and the host immune system.

This study reveals that combining rectal administration of Prevotella with oral RPP enhances cellular immune function and humoral immunity in mice. Specifically, pretreatment with RPP before injecting the inactivated PRRSV vaccine intramuscularly boosts immunity by modulating Prevotella_copri and increasing TDCA secretion. Wang noted that TDCA activates the IL-6/JAK1/STAT3 pathway, promoting the proliferation of normal gastric epithelial cells (GES-1) (38). Additionally, TDCA triggers IL-8 gene expression through Rel A phosphorylation, which attracts T cells to inflammation sites (39). Furthermore, dietary TDCA supplementation helps protect the intestinal mucosa by increasing resistance to apoptosis-induced injury, suggesting a protective role for TDCA in intestinal health (40). TDCA also enhances jejunal immunity and mucosal barrier function in piglets (41).

The immune function of the intestinal mucosa is vital for host health. Intestinal chyme metabolites act as signaling molecules and metabolic precursors, essential for maintaining immune homeostasis (41). Pretreating with RPP before administering the inactivated PRRSV vaccine intramuscularly effectively maintains this immune balance.

## 5 Conclusion

**Figure 10:**
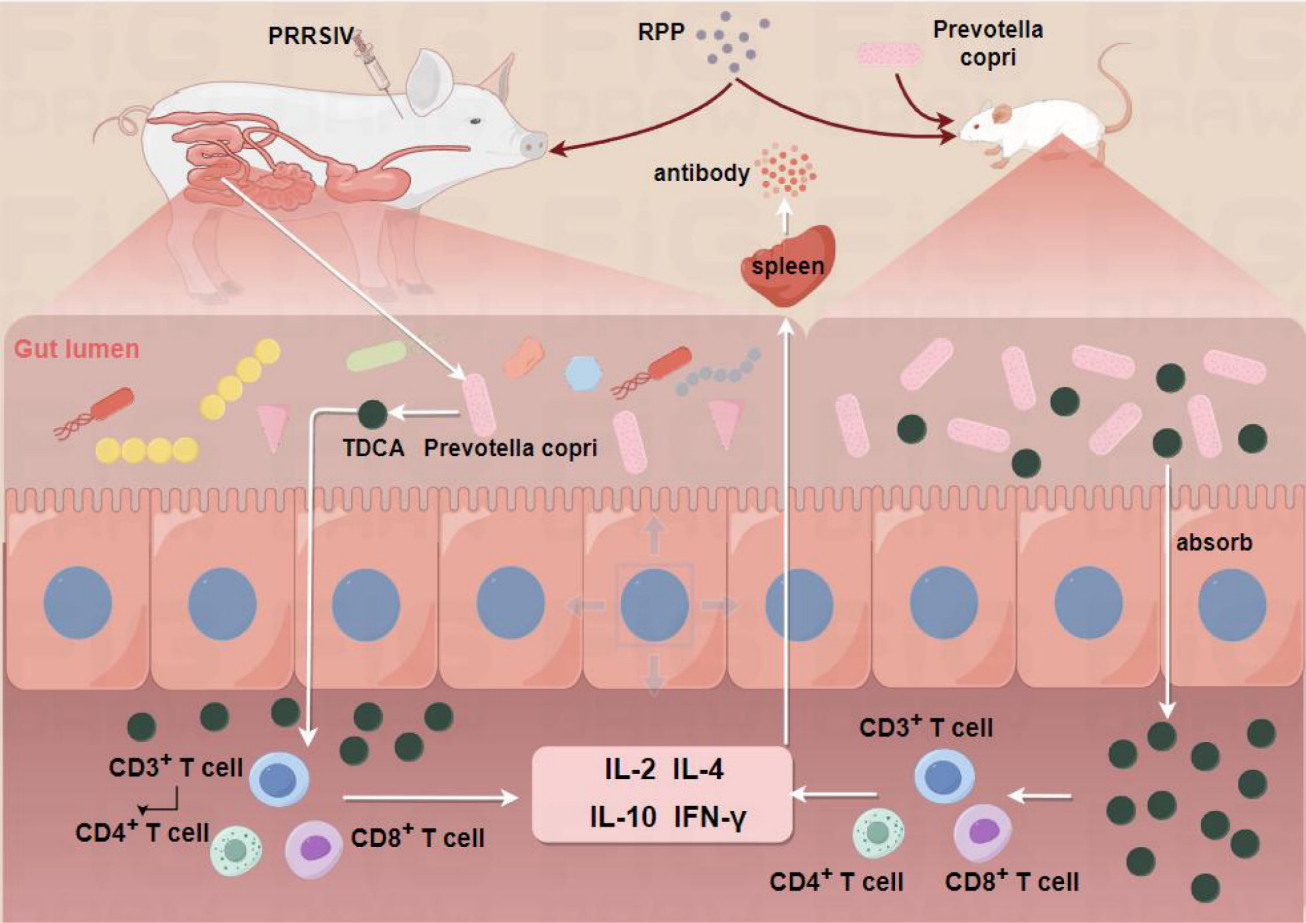
Pattern diagram of RPP improving inactivated PRRSV vaccine immune effect.

Administering 10 mg/kg of RPP orally before the inactivated PRRSV vaccine injection significantly improves vaccine efficacy. This improvement is mediated by key species and metabolites, notably Prevotella_copri and TDCA, which are induced by RPP (**Figure 10**).

## Data availability statement

The datasets presented in this study can be found in online repositories. The names of the repository/repositories and accession number(s) can be found in the article/**Supplementary Material**.

## Ethics statement

The animal study was approved by the medical ethics committee of Zhaofenghua Biotechnology (Fuzhou) Co. Ltd (NO. LL-2022-005). The study was conducted in accordance with the local legislation and institutional requirements.

## Author contributions

GX: Writing – original draft. ZY: Data curation, Writing – original draft. YZ: Writing – original draft, Data curation. QF: Writing – original draft, Data curation. LX: Writing – original draft, Data curation. SL: Writing – original draft, Data curation. SB: Writing – original draft, Resources. QW: Writing – review & editing, Project administration.
