# Supplementary figures and images for: Integrated microbiome and metabolome analysis reveals that new insight into Radix pseudostellariae polysaccharide enhances PRRSV inactivated vaccine
Source: Front Immunol. 2024 Jun 26;15:1352018. doi: 10.3389/fimmu.2024.1352018 (PMC11233517; doi:10.3389/fimmu.2024.1352018)

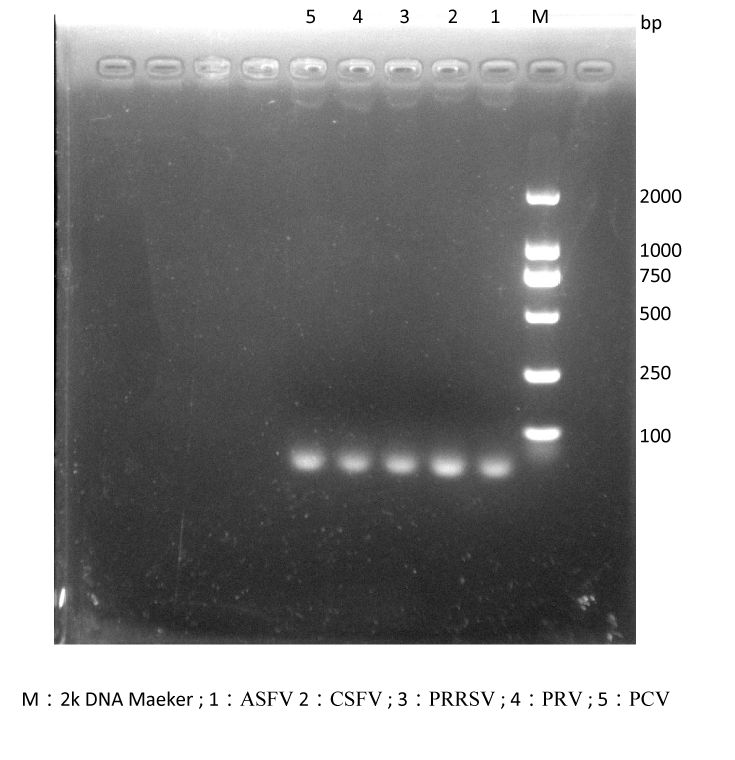

Supplement: Supplementary file 1 [file Image_1.tif]

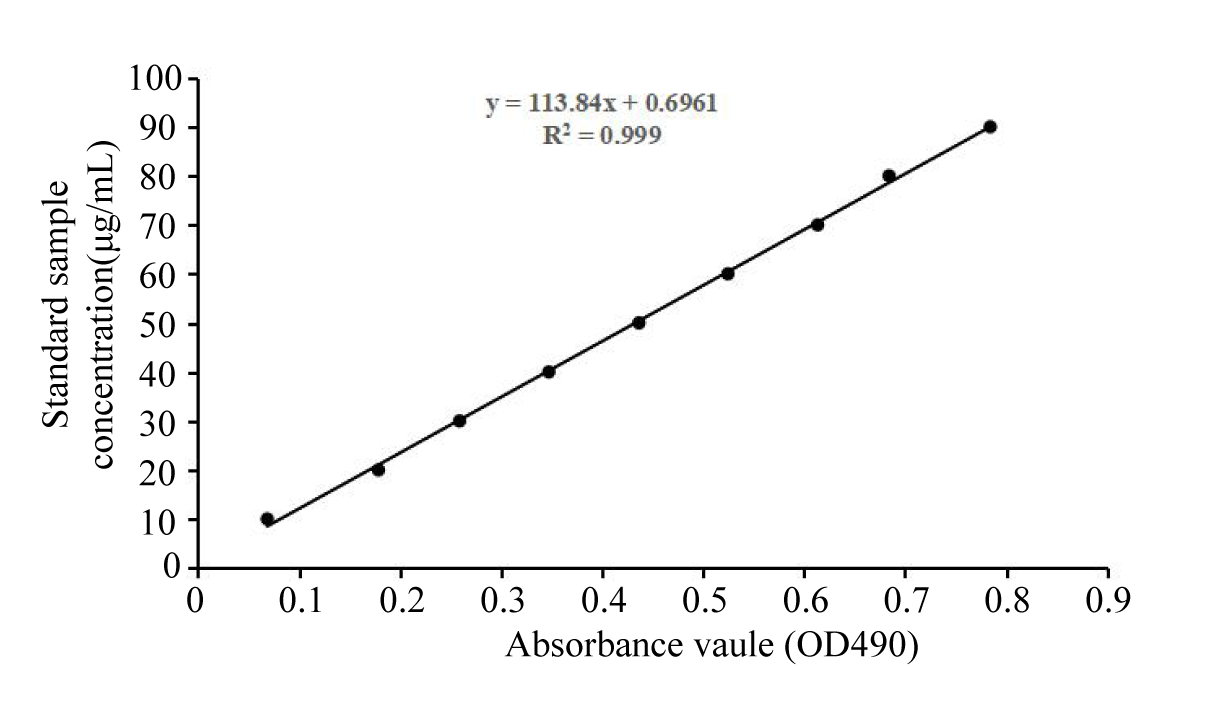

Supplement: Supplementary file 2 [file Image_2.tif]

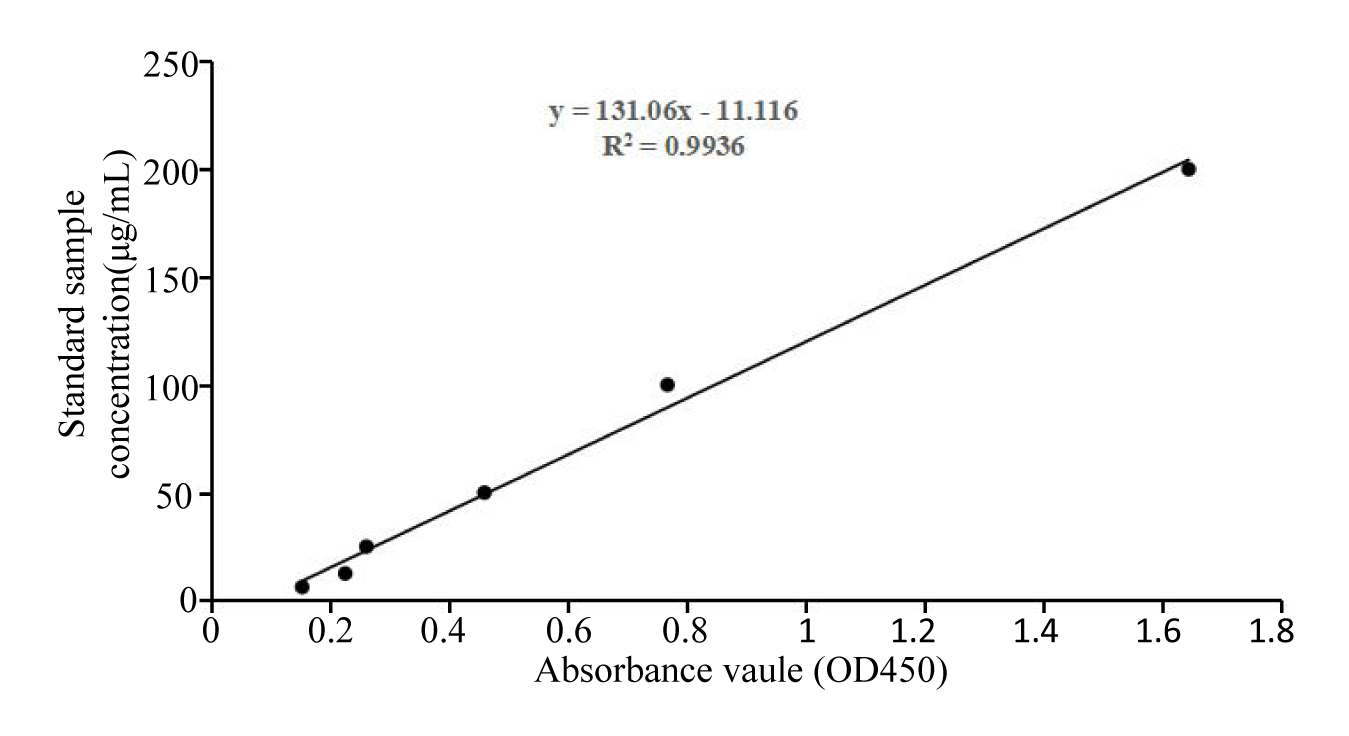

Supplement: Supplementary file 3 [file Image_3.tif]

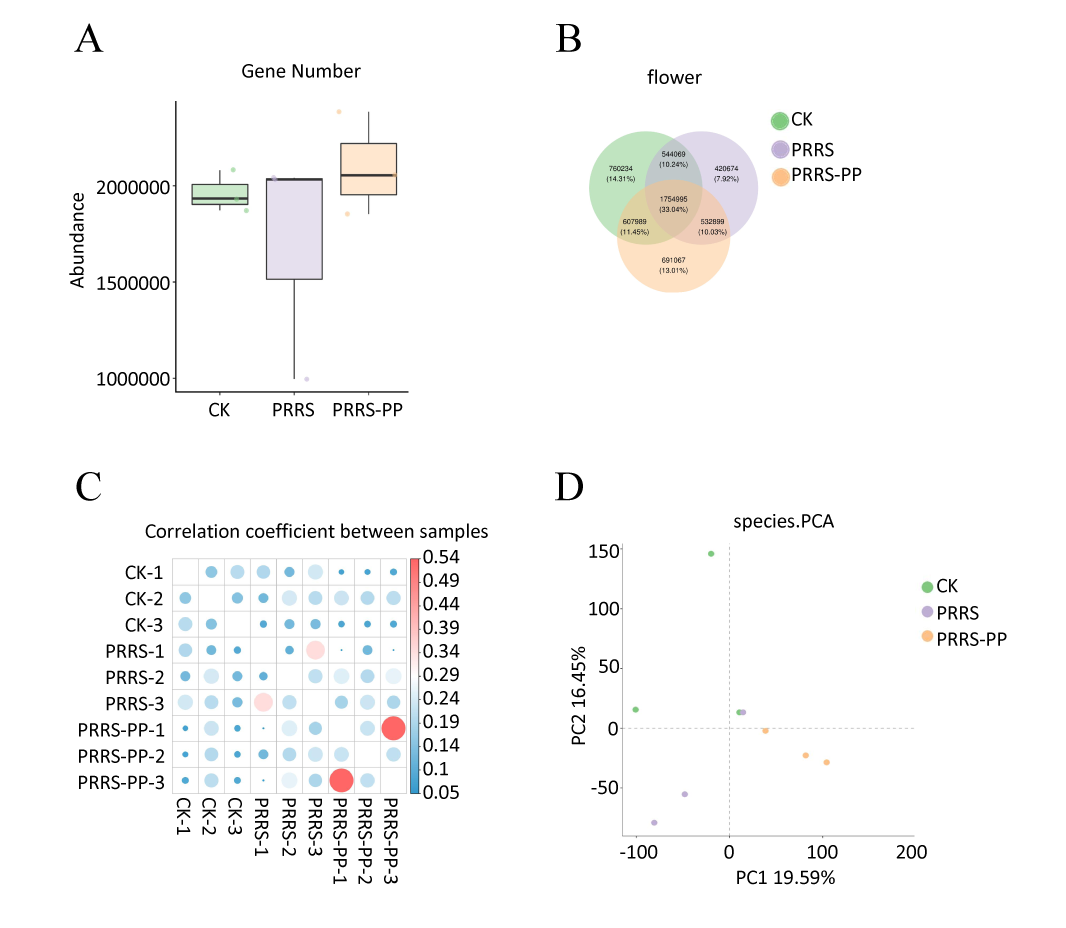

Supplement: Supplementary file 4 [file Image_4.tif]

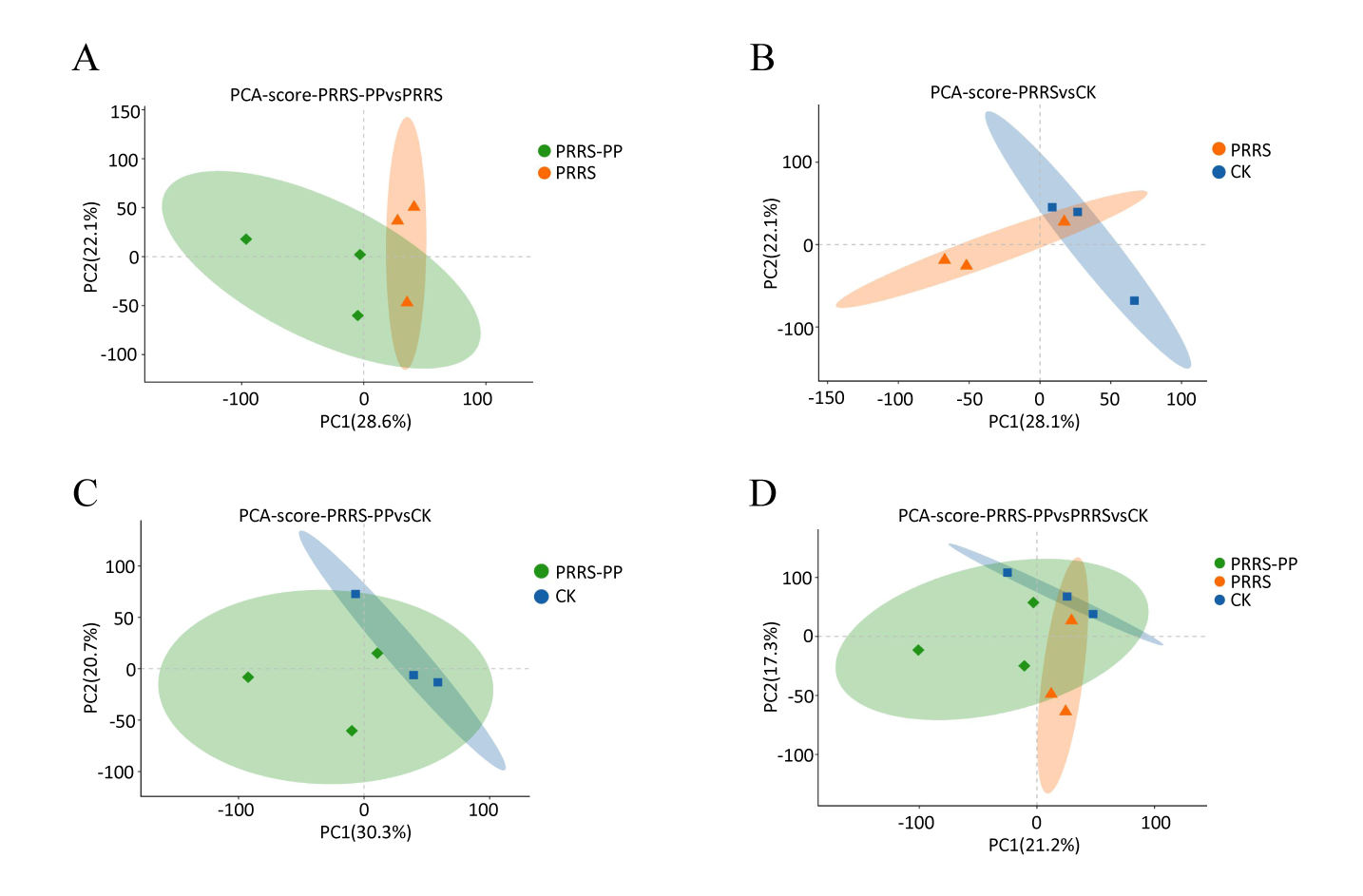

Supplement: Supplementary file 5 [file Image_5.tif]
