# Supplementary material for: Integrated microbiome and metabolome analysis reveals that new insight into Radix pseudostellariae polysaccharide enhances PRRSV inactivated vaccine
Source: Front Immunol. 2024 Jun 26;15:1352018. doi: 10.3389/fimmu.2024.1352018 (PMC11233517; doi:10.3389/fimmu.2024.1352018)
Supplement: Supplementary file 6 [file DataSheet_1.pdf]

Prokaryotic expression of Gp5 protein

Then recombinant plasmid pET-32a-Gp5 was constructed in our previous study, and was transfected into BL21 competent cells. Then cells were induced expression with 1mM IPTG at 37°C for 6h. The target protein was purified by nickel affinity chromatography, and its protein concentration was determined by BCA method.

Results

The result of SDS-PAGE showed that the target Gp5 protein was induced expression (Fig1). The purified Gp5 protein concentration was 7.53μg/μL tested by BCA (Tab1).

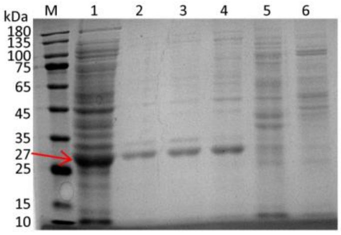

Fig1： Induced expression of Gp5 target protein

M:Marker; 1:Unpurified protein; 2-4:Target protein(27kDa): 5-6:Effluent

Tab1:BCA method for detecting protein concentration

Number	BSA standard(2μg/μL）	Target protein(μL)	Sample buffer(μL)	Total volume	Protein content	OD value
0	0	0	20	20	0	0.156800002
1	1	0	19	20	2	0.196299999
2	2	0	18	20	4	0.229800001
3	4	0	16	20	8	0.280099988
4	8	0	12	20	16	0.39410001
5	12	0	8	20	24	0.495499998
6	16	0	4	20	32	0.58859998
7	20	0	0	20	40	0.701900005
8	0	20	0	20	7.53	0.270700008
